# Per- and polyfluoroalkyl substances (PFAS) in fish collected from the Rio Grande and reservoirs in northern New Mexico

**DOI:** 10.1371/journal.pone.0336856

**Published:** 2025-11-19

**Authors:** Justin Clements, Jenna Stanek, Cyler Conrad, Jessica Celmer, Hanna Mora, Zachary Jones, Kylie Gallegos, Chauncey Gadek, Shannon Gaukler

**Affiliations:** 1 Environmental Protection and Compliance Division, Environmental Stewardship Group, Los Alamos National Laboratory, Los Alamos, New Mexico, United States of America; 2 Earth Systems Science Division, Risk and Environmental Assessment Group, Pacific Northwest National Laboratory, Richland, Washington, United States of America; 3 Department of Anthropology, University of New Mexico, Albuquerque, New Mexico, United States of America; 4 Department of Biology, University of New Mexico, Albuquerque, New Mexico, United States of America; US Geological Survey Columbia Environmental Research Center, UNITED STATES OF AMERICA

## Abstract

Per- and polyfluoroalkyl substances (PFAS) are a group of industrial and commercial chemicals widely used throughout the world due to their beneficial chemical properties. Because of their widespread use, their chemical stability, and their ability to be transported over long distances through atmospheric deposition and movement through waterways, PFAS are found throughout most aquatic ecosystems; yet large sampling gaps exist among reservoir and river ecosystems in the desert southwest of the United States. In this study, we examine PFAS concentrations in the tissue of fish (catfish [channel and blue], common carp, smallmouth bass, northern pike, walleye, white crappie and white sucker) collected in northern New Mexico, including examining PFAS composition and concentration relative to trophic level distribution. We collected fish from two man-made reservoirs and from the Rio Grande. We then collected muscle and liver tissues from fish specimens, which were screened for 39 PFAS compounds. We detected PFAS compounds in most fish tissue sampled, including the biomagnification of PFAS compounds within liver samples, with PFOS concentrations ranged from 1.13 to 350.1 (64.4 average) times higher in the liver samples compared to muscle samples. Most PFAS concentrations within muscle samples were within the range of atmospheric transportation previously reported and average tissue concentrations of PFAS were calculated to be 2.02 ± 1.81 ng g^-^1. Using stable isotopes as a predictor of trophic-foraging exposure and PFAS concentrations, we noted a correlation between enriched δ^15^N values, which had higher perfluorodecanoic acid concentrations.

## Introduction

Per- and polyfluoroalkyl substances (PFAS) are a group of synthetic organofluorine chemicals. These man-made chemicals encompass approximately 15,000 different chemical structures [1]. Although the simplest PFAS compound, carbon tetrafluoride, was discovered in 1886 [2], the discovery of mainstream PFAS compounds is credited to the accidental discovery of Teflon™ by Roy J. Plunkett at DuPont in 1938 [3]. PFAS have become commercially prevalent since the 1940s and, with their mass production, have been incorporated into numerous everyday products [4]. The chemical properties that once made PFAS a commercial success, including their hydrophobic and stability properties, have resulted in environmental concerns.

The unique chemical properties of PFAS (namely their nonstick nature, degradation resistance, stability, and heat tolerance) have resulted in their use in many common applications. PFAS can be found in firefighting foams (predominantly in aqueous film-forming foam [AFFF]), paints, textiles, personal care products, food packaging, nonstick cookware and numerous other commercial products. PFAS are comprised of a carbon backbone in which hydrogen atoms are replaced by fluorine atoms, generating a carbon-fluorine bond. This polar covalent bond is one of the strongest bonds in chemistry. Because of their extraordinary stability, PFAS have been labeled as “forever chemicals” [5]. Additionally, PFAS are found in most natural environments because of their stability, ease of transportation, and widespread use. The ubiquity of PFAS compounds within the environment has been the result of unintentional and intentional commercial release. PFAS can be released into the environment through the use of everyday products and activities, including the application of sunscreen or cosmetics and the use of toilet paper, all of which discharge PFAS through wastewater effluent that ends up in natural environments [6]. Travis et al., 2024 found PFAS in water samples taken from all major rivers in New Mexico (including samples from the Rio Chama and Rio Grande) [7]. Additionally, a study conducted in the Rock River in Illinois found high levels of PFAS compounds, with the highest concentrations sampled next to urban and industrial locations. Interestingly, the predominant PFAS compound within the samples was not consistent from year to year [8]. This was further demonstrated in the Rio Grande near Albuquerque New Mexico [9]. Beisner et al. 2024 demonstrated that PFAS concentrations increased within urban areas by an order of magnitude and that concentration of PFAS compounds shift over time in the Rio Grande [9].

Although originally thought to be an inert compound with little to no environmental impact or health effects, widespread research has now demonstrated that they are, in fact, harmful. The health effects of PFAS exposure have been known by chemical corporations as early as the 1970s, but these effects were not publicly recognized until the 1990s. Currently, many PFAS have been shown to possess carcinogenic properties, cause thyroid disease, and affect pregnancy and birth rates, as well as multiple other health effects [10,11]. New PFAS compounds are developed annually and incorporated into consumer products. Many of these newer compounds are short-chain (C ≤ 8) PFAS that have not gone through toxicological studies, and the health effects are unknown for some of the short-chain compounds [12]. Not all compounds within the PFAS group will have the same health effects or the same chemical properties. For example, research demonstrates that some short-chain PFAS compounds have more significant hepatotoxicity (liver) than long-chain PFAS [12]. For this reason, it is important to understand PFAS composition and concentrations when examining PFAS toxicity in animals, humans, and the natural environments.

PFAS are known to be transported through waterways; therefore, a critical health concern regarding PFAS is the consumption of contaminated food and water. One major route of human PFAS exposure is through the ingestion of contaminated fish [13–15]. Fish accumulate PFAS compounds through ingestion and respiration while swimming in bodies of water that are polluted with PFAS compounds. Figueroa-Muñoz et al. 2025 identified that perfluorooctanesulfonic acid (PFOS) accumulates in the skin of fish over time, and by removing the skin before consumption, PFOS ingestion rates significantly decrease [16]. A study conducted by Barbo et al. (2023) estimated that the median total PFAS concentrations within fish muscle in the United States (U.S.) was 9.5 ng g^-1^ (nanograms per gram), with the highest concentration observed being 286.8 ng g^-1^ [13]. When evaluating PFAS concentrations across different fish species, a study conducted in the U.S. Great Lakes by Miranda et al. (2023) found that PFOS was the predominant PFAS compound from PFAS analyzed across all fish taxa and that PFOS biomagnified between trophic levels, with lower concentrations found in prey fish versus predatory fish, while other long-chain PFAS compounds biomagnified factors depended on fish species [17]. These findings are supported by Barbo et al. 2023 and Wathen et al. 2025 who also found high levels PFOS and other long-chain PFAS compounds in fish tissue [13,18]. The Great Lakes Consortium for Fish Consumption Advisories published guidelines in 2019 that suggest unrestricted consumption of fish should contain PFOS lower than 10 ng g^-1^ [19].

In this current study, our primary aim was to examine PFAS concentrations in fish tissue collected from bodies of water in northern New Mexico, including a river (Rio Grande) and two manmade reservoirs (Abiquiu and Cochiti) to monitor PFAS concentration in fish commonly consumed from this geographical area. All three water bodies have a diversity of fish species that are consumed by humans. Specifically, we sought to classify PFAS composition and concentrations in fish tissue (muscle and liver). We also sought to examine the similarities and differences between PFAS composition and concentrations between tissue type (muscle vs. liver) and how dietary stable isotopes collected from the fish liver samples are related to PFAS concentrations. Stable isotopes can be used as a predictor of trophic-foraging exposure.

## Materials and methods

### Site description

**Fig 1:**
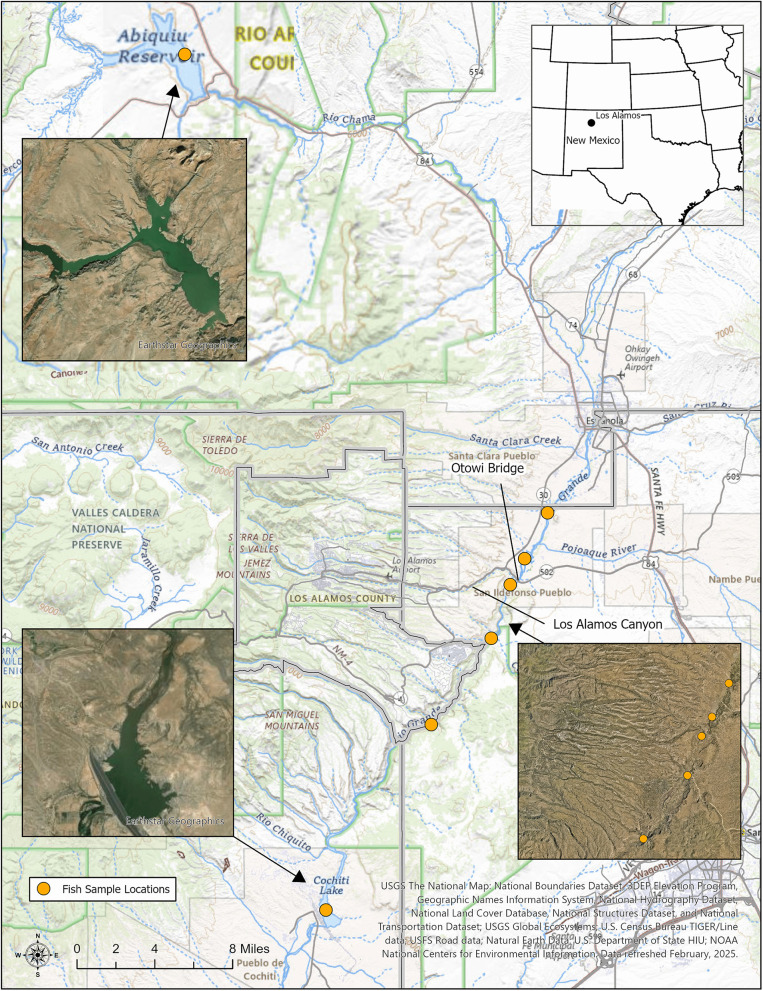
Fish sampling points across the three site locations (Abiquiu and Cochiti reservoir and the Rio Grande). Figure produced by Institution GIS at Los Alamos National Laboratory.

We collected samples from three bodies of water in northern New Mexico, including a river (Rio Grande) and two man-made reservoirs (Abiquiu and Cochiti) (Fig 1). The Rio Grande flows through Colorado, New Mexico, Texas, and Mexico. Water from the river is used as irrigation for agriculture, for recreation (boating and rafting), and as drinking water for animals and humans, providing water to approximately 6 million people [20]. The Rio Grande is the primary input and output of Cochiti Reservoir, whereas the Rio Chama primarily feeds Abiquiu Reservoir. Both reservoirs in this study were created through the construction of large, anthropogenic dams and are home to multiple freshwater fish taxa.

### Sample collection

Sample collection from the two reservoirs and a segment of the Rio Grande (Fig 1) was conducted in the summer of 2023 under New Mexico Department of Game and Fish (NMDGF) permit #2864.

We collected fish samples throughout both Cochiti Reservoir (*n *= 10) and Abiquiu Reservoir (*n *= 10) using gillnets and by rod-and-reel and hoop nets from five location in the Rio Grande (*n *= 20). Samples were collected as a part of a larger monitoring program which dictated available sample size approved at each location. All available samples were used within the data analysis. No specific species was targeted during this sampling event, however, collected species were those that could be used for human consumption. We set the gillnets and hoop nets in the late afternoon and checked the following morning, removing fish from nets and placing them in a livewell. We euthanized the fish using an Ikigun under Institutional Animal Care and Use Committee approved (Protocol 23–74) euthanasia methods. The number of fish collected and euthanized was dictated by monitoring program procedures. All remaining captured fish were released at the sampling locations. Fish taxa that we collected in the reservoirs included common carp (*Cyprinus carpio, n* = 3), smallmouth bass (*Micropterus dolomieu, n* = 3), catfish (*Ictalurus punctatus or Ictalurus furcatus, n* = 3), walleye (*Sander vitreus, n* = 4), white sucker (*Catostomus commersonii, n* = 1), northern pike (*Esox Lucius, n* = 3), and white crappie (*Pomoxis annularis, n* = 1). Fish taxa collected from the Rio Grande included common carp (*n* = 2), blue catfish (*n* = 1), channel catfish (*n* = 16), and white sucker (*n* = 1). Fish were categorized into two types of feeding strategies: bottom or predator feeders. The bottom feeders predominantly displayed a flat ventral region (catfish and white suckers) with downward facing mouth parts (catfish, carp, white sucker) while the predator feeders predominantly displayed active hunting strategies and consume other fish and aquatic animals (bass, crappie, northern pike, and walleye). We processed fish samples at the collection site the same day and collected muscle (fillet, used for human consumption) and liver samples from each fish. Fish mass and length were recorded (S1 Table), however, we did not to determine the sex of the fish. Equipment used in the collection of tissue samples had previously been screened for PFAS contamination. Further, staff were instructed not to wear PFAS ladened cosmetics, new clothing, or handle fast food wrappers. After fish were euthanized, they were transported to a clean processing area. All staff members who processed fish wore PFAS-free gloves and safety glasses. Fish samples were placed on a clean PFAS free cutting board. The skin and scales were removed from the side of the fish with a clean knife. A filet was then removed from the fish avoiding any additional connective tissue or scales. The fish’s abdominal cavity was opened; the liver was located and removed. Samples were immediately placed into their own individual PFAS-free bottles; labeled, sealed with chain-of-custody tape, stored on ice, and shipped to GEL Laboratories for analysis. A separate sample of liver was collected for stable Isotope analysis. PFAS water blanks were collected at the same time that the fish muscle and liver samples were collected to assess potential contamination. Briefly, a PFAS-free water sample was transported to the field, open during the sampling event, and poured into a secondary PFAS free bottle, sealed and sent off for PFAS analysis.

Samples used for PFAS analysis were shipped under full chain of custody to the analytical laboratory, GEL (Charleston, South Carolina), and analyzed for 39 PFAS, listed in Table 1. Sample analyses were conducted using methodology outlined in GEL Laboratories, LLC (GEL), an EPA (Environmental Protection Agency) and DOECAP (Department of Energy Consolidated Audit Program) accredited laboratory, standard operating procedures. The standard operating procedure utilizes an EPA approved liquid chromatography tandem mass spectrometry LC-MS/MS method to quantify PFAS concentrations in aqueous, solid, biosolids, and tissue samples [21]. Sample analysis for PFAS by LC-MS/MS was conducted on an AB SCIEX high performance liquid chromatograph coupled to a triple quadrupole mass spectrometer with manufacturer software. Tissue samples were homogenized and 0.5 gram aliquot of a sample was loaded onto the solid phase extraction cartridge for analysis. GEL Laboratory’s quality control samples include method and instrument blanks, laboratory control samples, matrix spikes (MS), and matrix spike duplicates (MSD), of approximately 40 PFAS compounds (MS and MSD), extracted internal standards, non-extracted internal standards, a sample duplicate, and calibration verification standards. Results of these laboratory quality control samples can be found on Intellus New Mexico website using advanced search Analytical Lab Quality Control Data option [22]. Concentrations of PFAS targets were quantified based on a comparison to the extracted internal standard. GEL Laboratory quantified method detection limits/ limit of detection (MDL/LOD) and practical quantitation limit/limit of quantification PQL/LOQ, along with internal standard area summaries for each sample and PFAS compound can be found at Intellus New Mexico data packages (COC# 2023 – (773, 812, 814, 843, 847, 857, 931, 932, 1013)). MDL/LOD and PQL/LOQ ranged for each PFAS compound examined but are included in the Intellus New Mexico reports along with reported detection limits, validation qualifiers (J-, NQ, R, U, UJ) and validation reason codes, and dilution factors. Specifically, calibration verification standards were not met for the rejected data, however, the target analytes were not detected in those samples and therefore not present in the sampled media. To conduct the statistical analysis the rejected data listed as non-detect and non-detects values were set to zero. GEL Laboratory provided data qualifiers for each sample analyzed and each report underwent secondary validation. GEL Laboratory provided data qualifiers for each sample analyzed and each report underwent secondary validation at LANL. The secondary validation procedures and level 4 (EPA) data packages for these COCs can be found on Intellus New Mexico website [22].

**Table 1:** Per- and polyfluoroalkyl substances examined within fish tissue listed alphabetically.

Per- and Polyfluoroalkyl Substances	Abbreviation
11-Chloroeicosafluoro-3-oxaundecane-1-sulfonic acid	11-Cl-PF3
1H, 1H, 2H, 2H-Perfluorodecane sulfonic acid	8:2 FTS
1H, 1H, 2H, 2H-Perfluorohexanesulfonic acid	4:2 FTS
1H, 1H, 2H, 2H-Perfluorooctane sulfonic acid	6:2 FTS
3-Perfluoroheptyl propanoic acid	7:3 FTCA
3-Perfluoropentyl propanoic acid	5:3 FTCA
3-Perfluoropropyl propanoic acid	3:3 FTCA
4,8-Dioxa-3H-perfluorononanoic acid	ADONA
9-Chlorohexadecafluoro-3-oxanonane-1-sulfonic acid	9-Cl-PF3O
Ethyl perfluorooctane sulfonamido ethanol[N-]	NEtFOSE
Ethyl perfluorooctane sulfonamido acetic acid[N-]	NEtFOSAA
Ethyl perfluoro-1-octanesulfonamide[N-]	NEtFOSA
Hexafluoropropyleneoxide dimer acid	HFPO-DA
Methyl perfluorooctane sulfonamido ethanol[N-]	NMeFOSE
Methyl perfluorooctanesulfonamidoacetic acid[N-]	NMeFOSAA
Methylperfluoro-1-octanesulfonamide[N-]	NMeFOSA
Nonafluoro-3,6-dioxaheptanoic acid	NFDHA
Perfluoro(2-ethoxyethane) sulfonic acid	PFEESA
Perfluoro-1-heptanesulfonic acid	PFHpS
Perfluoro-1-octanesulfonamide	PFOSA
Perfluoro-4-methoxybutanoic acid	PFMBA
Perfluorobutanesulfonic acid	PFBS
Perfluorobutanoic acid	PFBA
Perfluorodecane sulfonate	PFDS
Perfluorodecanoic acid	PFDA
Perfluorododecanesulfonic acid	PFDoS
Perfluorododecanoic acid	PFDoA
Perfluoroheptanoic acid	PFHpA
Perfluorohexanesulfonic acid	PFHxS
Perfluorohexanoic acid	PFHxA
Perfluorononanesulfonic acid	PFNS
Perfluorononanoic acid	PFNA
Perfluorooctanesulfonic acid	PFOS
Perfluorooctanoic acid	PFOA
Perfluoropentanesulfonic acid	PFPeS
Perfluoropentanoic acid	PFPeA
Perfluorotetradecanoic acid	PFTeDA
Perfluorotridecanoic acid	PFTrDA
Perfluoroundecanoic acid	PFUnA

We collected liver samples from 38 of the 40 fish sampled (Abiquiu *n* = 10; Cochiti *n* = 8; Rio Grande *n* = 20). We did not collect the liver of two fish samples as there was concern with the deterioration of the liver sample during removal. We cut each individual fish liver using a fillet knife to create two separate samples: a PFAS sample and a stable isotope sample. Stable isotopes were examined in the liver samples as we presumed that there would be higher concentrations of PFAS found within this specific tissue to compare with the isotopic findings. We placed the samples on ice for transport from the sampling locations. Samples designated for isotopic analysis remained frozen in a −20°C laboratory freezer for approximately six months until transported to the University of New Mexico, Center for Stable Isotopes (UNM-CSI), Albuquerque, New Mexico. UNM-CSI lipid extracted each liver sample then dried and homogenized the samples before weighing approximately 0.5–0.6 mg of tissue into tin capsules for carbon (δ^13^C) and nitrogen (δ^15^N) stable isotope analysis. Analytical laboratory personnel used a Costech 4010 elemental analyzer (Valencia, CA) coupled to a Thermo Scientific Delta V Plus isotope ratio mass spectrometer (Bremen, Germany) at UNM-CSI to measure stable isotope samples. Isotope data are reported in delta notation (δ) as parts per thousand (‰; or per mil) where δ = (*R*_sample_/*R*_standard_ − 1) × 1000, where *R*_sample_ and *R*_standard_ are the relative ratios of the heavy and light isotopes (^13^C/^12^C or ^15^N/^14^N) in a sample and standard, respectively. δ^13^C and δ^15^N values are referenced against the international standards Vienna Pee-Dee belemnite (V-PDB) and atmospheric nitrogen (air; N_2_), respectively. Measured isotope values were calibrated against an international reference standard (USGS-40) using internal reference materials run alongside plasma samples to correct for instrument drift within and between analytical runs [23]. Repeated measurements of reference materials yielded an analytical precision of (±SD) of ± 0.2‰ for δ^13^C and δ^15^N. All sample C:N ratios were within acceptable ranges except sample SFB-23–289335 (see S1 Table), which had an elevated ratio; however, given our total sample size and the overlapping consistency in stable isotope values for SFB-23–289335 and all other fishes, we included that sample in all subsequent analyses.

### Statistical analysis

Analytical laboratory staff imported PFAS concentration data for the fish muscle and liver samples analyzed by GEL into Intellus New Mexico, a publicly available data portal [22]. Once uploaded by the analytical lab, we downloaded the data files directly from Intellus New Mexico, an open-source data repository to use in our statistical analyses. We generated heat maps of PFAS results in fish tissue using R Statistical Software 4.4.1 to visually assess results. Statistical differences in PFOS concentrations between tissue type (liver and muscle) and locations are generated using GraphPad Prism 10.4.1. We calculated differences in PFOS concentrations between feeding strategies using an unpaired t-test for normally distributed data (p < 0.05) or a Mann-Whitney test for non-normally distributed data (p < 0.05), whereas PFOS concentrations among locations is determined using one-way ANOVA for normally distributed data (p < 0.05) or a Kruskal–Wallis test for non-normally distributed data (p < 0.05). We generated percentages of PFAS compounds by concentration in GraphPad Prism 10.4.1. Only data with positive validation qualifiers were used in the heat map and PFOS analysis. We ran a hierarchical clustering analysis on stable isotopic data and fish taxa to visually assess the results in JMP version 16 [24].

In addition, we used nonmetric multidimensional scaling (NMDS) with a Bray-Curtis dissimilarity matrix to assess overall differences in PFAS composition (using PFAS concentrations) in fish (muscle and liver) at different locations (Abiquiu Reservoir, Cochiti Reservoir, and the Rio Grande). We used a separate NMDS to assess overall differences in PFAS composition in fish liver between the feeding strategies (predator versus bottom feeder) and stable isotope values. We determined the appropriate number of dimensions by plotting final stress versus the number of dimensions and chose the number of axes beyond which reductions in stress were small [25]. We added PFAS labels based on weighted average abundance across the sites. The location of a label can be thought of as its “optimum” in the NMDS space, so we could visually assess which PFAS are associated with which locations. Differences in locations and feeding groups were tested using analysis of similarities [26,27]. A separate NMDS analysis was used to evaluate overall differences in PFAS composition in fish liver between the feeding strategies (predator versus bottom feeder) and stable isotope values using a similar procedure, with the exception of adding PFAS labels of weighted average abundance. We investigated the variables that were driving the distribution patterns (p < 0.05) relative to feeding strategies based on stable isotope values using the R *envfit* function in the R *vegan* package v.2.6.8 [28] which uses a permutation test to evaluate significance and the direction and length of a vector. We also assessed the differential occurrence of PFAS detected in liver samples at all locations in feeding strategies (predator versus bottom feeder) with a simper analysis, which calculates the average contribution of each variable to the overall Bray-Curtis dissimilarity between groups, using the R *simper* function in the R *vegan* package [28].

To further examine the relationship between δ^15^N and PFAS compounds identified in the NMDS analysis, we modeled PFDA, PFUnA, and PFOS concentrations using a multivariate mixed-effects model in the R *brms* package [29]. Given that the PFAS concentrations included a high proportion of zero values (i.e., values below the detection limit or true absence), we specified a hurdle log-normal distribution family for the response variables. Liver δ^15^N values served as the sole continuous predictor. To account for species-level variation in δ^15^N, we included a random intercept for species.

We specified a weakly informative prior on all β coefficients (~N (0, 3)). For all other parameters, we applied the brms default priors as follows: (1) hurdle (zero-inflation) probabilities: β (1, 1) prior, which is uniform on the probability scale; (2) intercepts: Student-t distributions with 3 degrees of freedom, centered at a compound-specific mean (e.g., PFDA: −0.7, PFOS: 3.4, PFUnA: −1.1), and scale 2.5; (3) standard deviations of random effects and residuals: weakly informative Student-t (3, 0, 2.5) priors, constrained to be positive; (4) correlation matrices among random effects: Lewandowski–Kurowicka–Joe (LKJ) prior with a η prior of 1, corresponding to a uniform prior over correlation matrices. We examined the influence of prior choice on our model by generating and plotting conditional effects for prior predictive distributions for each response variable using the full model specification. The prior predictive checks suggested that our chosen priors were sufficiently uninformative, allowing a broad range of plausible values and relationships before incorporating the data. Based on these results, we proceeded with the specified priors for our analysis.

We ran the model for 10,000 iterations, thinned posterior samples by retaining every 10th iteration and discarded the first 5,000 iterations as burn-in. We evaluated model convergence and fit by ensuring that effective sample sizes of parameter estimates were > 1,000 and Rhat values were 1.0. We extracted and visualized model predictions with the mcmc_plot() and conditional_effects() functions from the R *brms* package. PFDA, PFUnA, and PFOS had higher than 20 percent positive detection rates within our dataset, allowing us to run a robust statistical analysis, whereas we excluded other PFAS compounds with lower than 20 percent detection rates from some analysis [30].

## Results

### PFAS concentrations in fish muscle

**Fig 2:**
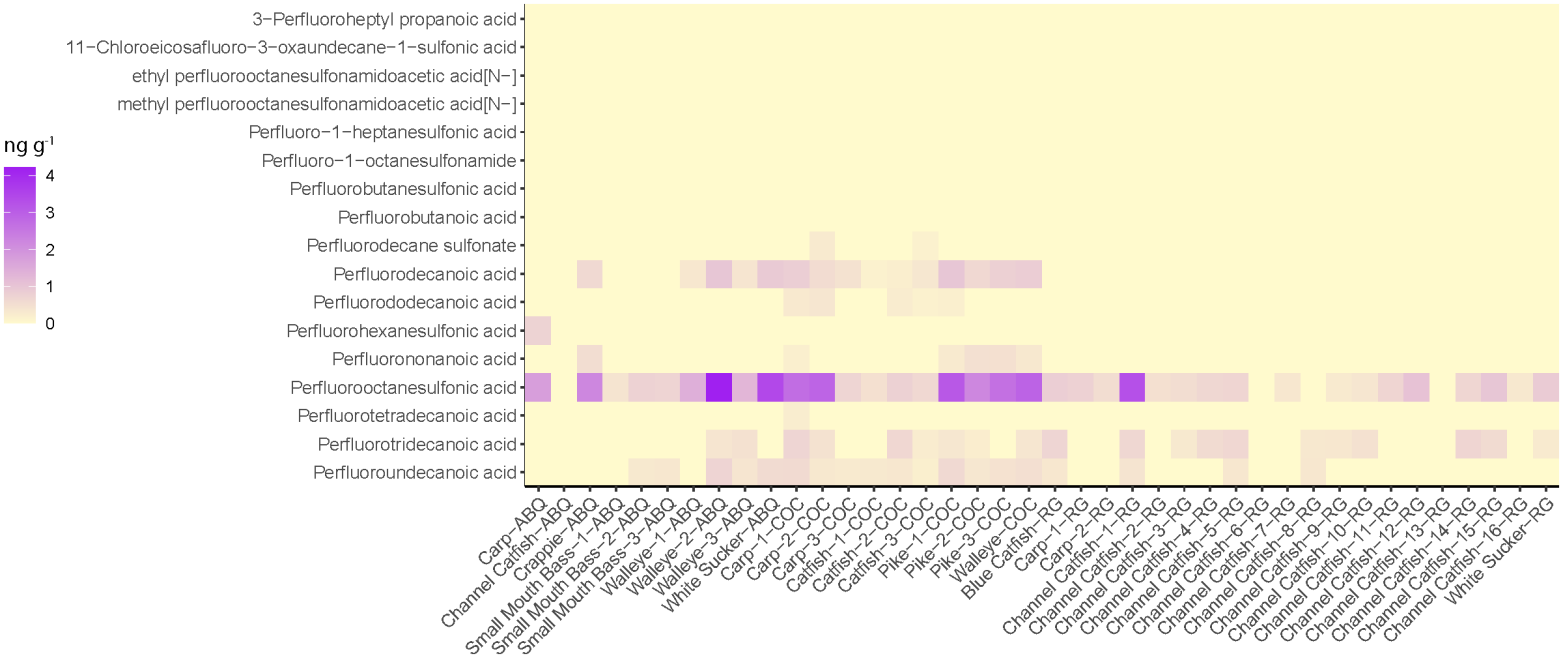
Heatmap of PFAS muscle tissue concentrations (ng g^-1^) from fish samples collected in Abiquiu (ABQ) and Cochiti (COC) Reservoirs and the Rio Grande (RG); PFAS displayed were detected at least once in either muscle or liver tissue.

We observed detectable levels of multiple PFAS compounds in 19 of the 20 fish muscle samples collected from Abiquiu and Cochiti Reservoirs (Fig 2). The PFAS compounds detected in fish muscle samples included PFHxS, PFOS, PFNA, PFDA, PFDS, PFUnA, PFDoA, PFTeDA, and PFTrDA and ranged from 95% detected for PFOS down to 5% detection for PFHxS, which was only detected in one fish collected from Abiquiu Reservoir. The three most common PFAS compounds detected in fish muscle samples from the reservoirs were PFOS (detected in 19 of the 20 fish muscle samples), followed by PFDA and PFUnA (both detected in 15 muscle samples). Within the fish tissue from both reservoirs, PFAS positive detection concentrations ranged from 0.169 ng g^-1^ to 4.22 ng g^-1^ within muscle samples, with the highest concentrations belonging to PFOS (range 0.414 to 4.22 ng g^-1^, mean of 1.87 ng g^-1^). The only fish tissue sample that did not have any PFAS detected in the muscle was a channel catfish collected from Abiquiu Reservoir. The majority of PFAS compounds were not detected in fish muscle in the Rio Grande (Fig 2). Only three PFAS compounds were observed: PFOS (85% detection rate), PFUnA (20% detection rate), and PFTrDA (60% detection rate); however, 18 of the 20 fish examined had at least one of these PFAS compounds detected within their muscle tissue. The two fish tissue samples that did not have any PFAS detected in the muscle tissue belong to two separate channel catfish collected from Rio Grande. All detectable levels of PFAS in the Rio Grande fish were below 1 ng g^-1^, with the exception of one tissue sample collected from a channel catfish, which had PFOS at a concentration of 3.28 ng g^-1^.

### PFAS concentrations in fish liver

**Fig 3:**
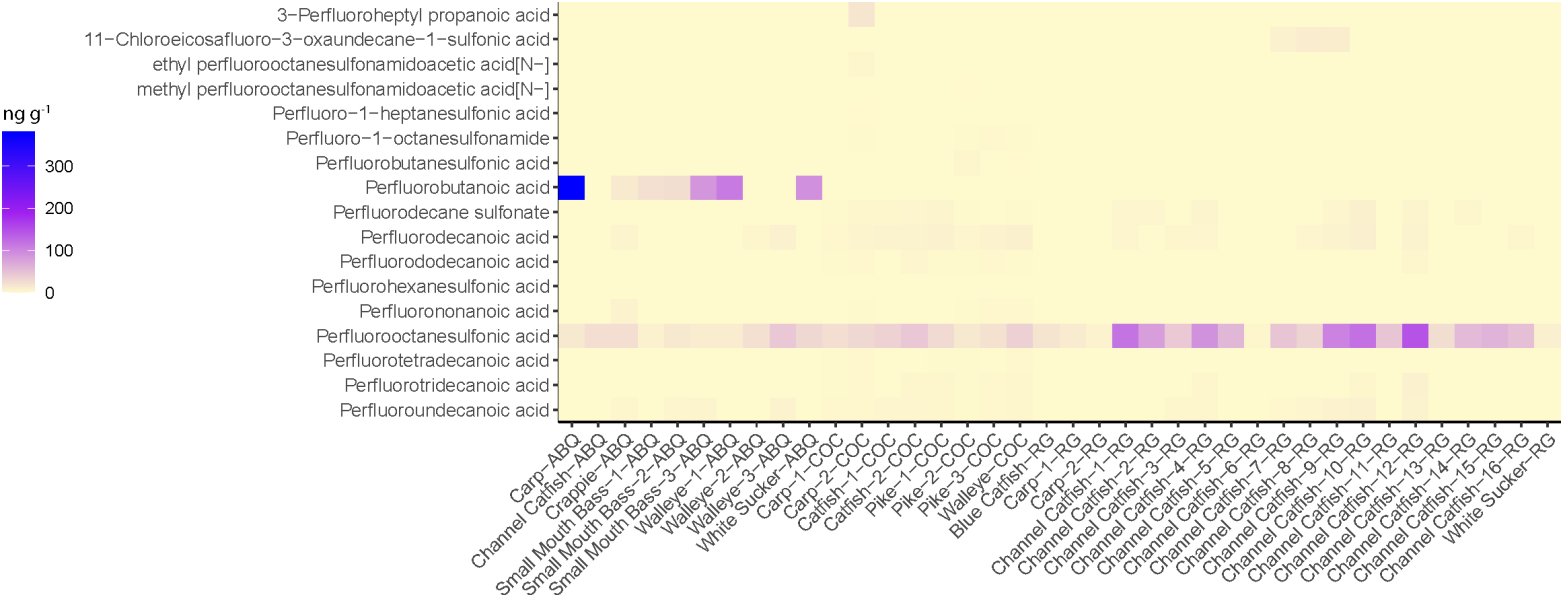
Heatmap of PFAS liver tissue concentrations (ng g^-1^) from fish samples collected in the Rio Grande (RG) and Abiquiu (ABQ) and (COC) Cochiti Reservoirs; PFAS displayed were detected at least once in either muscle or liver tissue.

We observed detectable levels of multiple PFAS compounds in 38 of the 38 fish liver samples collected from Abiquiu and Cochiti Reservoirs and the Rio Grande (Fig 3). Liver samples were collected from the same fish that were used for the muscle analysis (38 out of 40 fish; we were unable to collect two liver samples). Positive PFAS detections in liver samples ranged from 0.326 to 382 ng g^-1^ and encompassed 16 different PFAS compounds (PFBA, PFBS, PFHpS, 7:3 FTCA, NEtFOSAA, NMeFOSAA, PFOSA, PFNA, PFOS, 11-Cl-PF3, PFDS, PFDA, PFUnA, PFDoA, PFTrDA, and PFTeDA; Fig 3). The highest PFAS concentration detected was collected from a common carp from Abiquiu Reservoir. The liver sample from the common carp had a concentration of 382 ng g^-1^ of PFBA (short-chain PFAS). The most commonly detected PFAS compound in fish liver samples was PFOS, which was detected in all of the samples examined (Fig 3). Samples collected from Abiquiu Reservoir also demonstrated a high abundance (concentrations and number of detections) of PFBA (short-chain PFAS) that was not seen in the fish collected from the Rio Grande or Cochiti Reservoir (Fig 3).

### PFOS in muscle and liver samples

**Fig 4:**
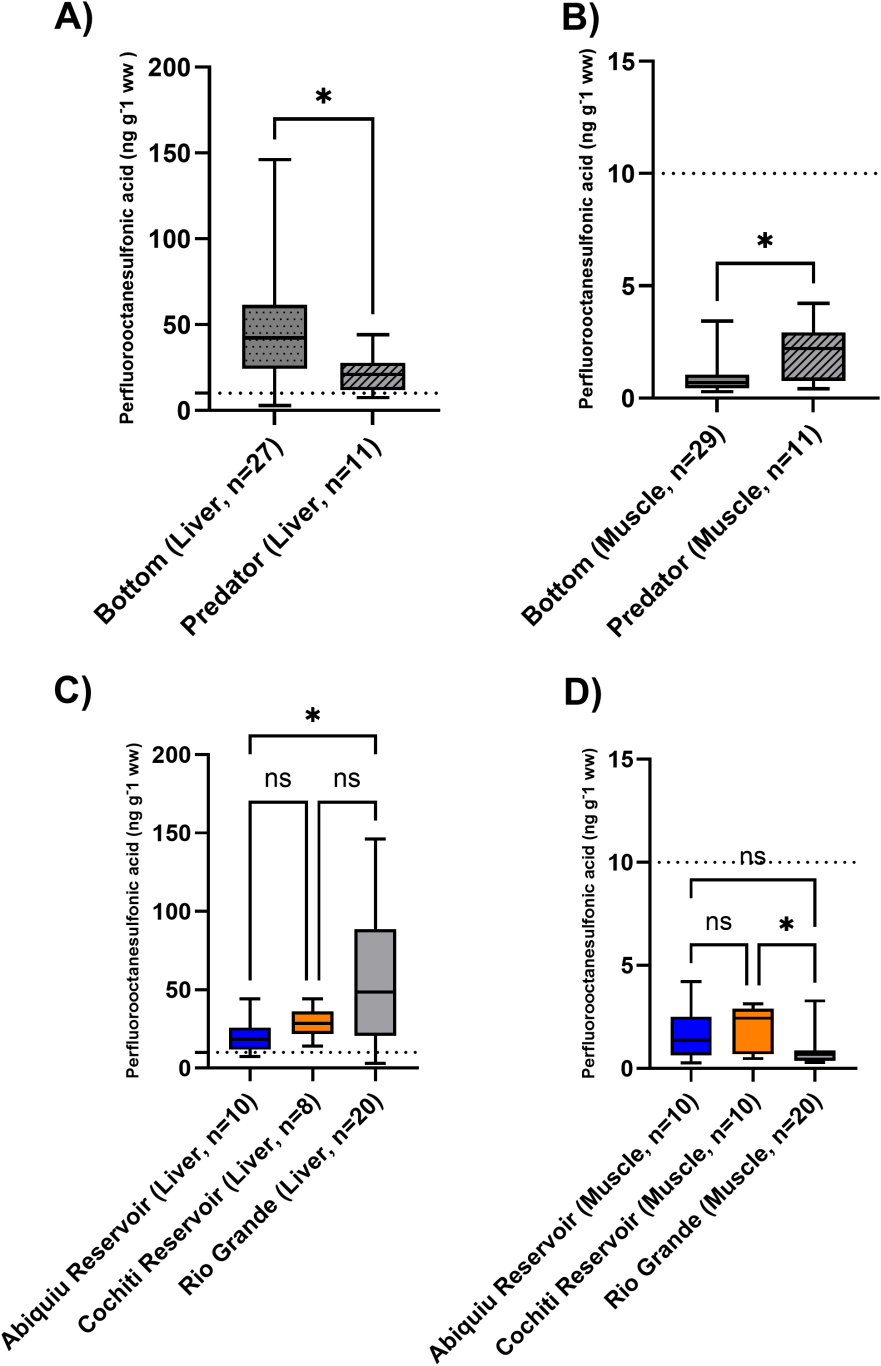
Comparison among PFOS concentrations in (A) liver samples collected from bottom-feeding and predator fish; (B) muscle samples collected from bottom-feeding and predator fish; (C) liver samples collected from Abiquiu and Cochiti reservoirs and the Rio Grande; and (D) muscle samples collected from Abiquiu and Cochiti reservoirs and the Rio Grande. Box plots and associated means with attached asterisk letters represent a significant difference (p = 0.05). Note the fish consumption limit of 10 ng g^-1^ represented by a dashed horizontal line on all graphs. Within section C) and D) ns refers to non-significant.

PFOS was ubiquitously found throughout most tissue sampled and across locations—unlike other PFAS—which allowed us to comprehensively examine among tissue types and sampling locations. When we examined PFOS composition and concentrations between muscle and liver samples from fish collected in the Rio Grande and reservoirs, the average PFOS concentrations were higher in liver (average 41.21 ± standard deviation 34.77 ng g^-1^) samples when compared with muscle samples (1.31 ± 1.09 ng g^-1^, Mann-Whitney test, U = 4, Sum of rates 824, 2257, p < 0.0001). PFOS was the predominant PFAS compound in both tissue types and was found at all sampling locations. Concentrations of PFOS ranged from 2.94 ng g^-1^ to 146 ng g^-1^, with a mean concentration of 41 ng g^-1^ (Fig 4) in liver tissue. Within liver tissue samples, most (35 of 38 liver samples) PFOS concentrations were above the 10 ng g^-1^ published fish consumption advisories guidelines, while all muscle samples fell below these guidelines (Fig 4). These results suggest bioaccumulation of PFOS in liver tissue from these sites. PFOS liver concentrations were significantly higher in bottom-feeding fish (mean of 49.3 ± 37.89 ng g^-1^) when compared with that of predatory fish (mean of 21.3 ± 11.22 ng g^-1^; unpaired t-test, t = 2.393, df = 36, p = 0.022); however, we observed the opposite trend within the muscle samples (Fig 4). A significant difference was also apparent in mean PFOS concentrations among different sampling locations in the liver samples, with PFOS being significantly higher in liver samples collected from the Rio Grande when compared with Abiquiu Reservoir (one-way ANOVA, df = 37, F stat 5.134, p = 0.011; Fig 4).

### PFAS profile comparisons

**Fig 5:**
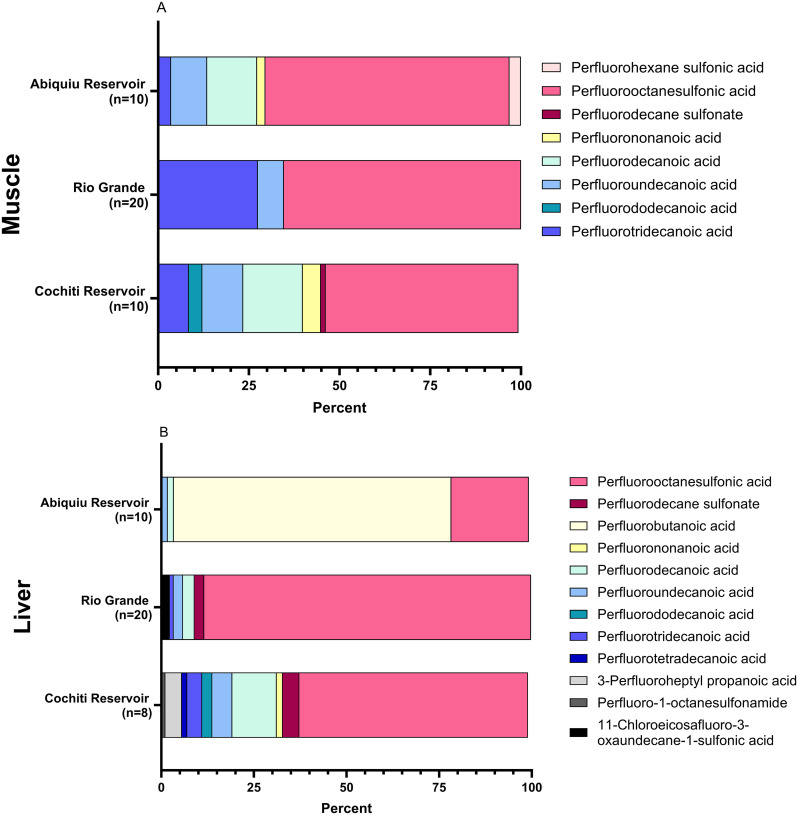
Percentage of PFAS compounds by concentration (only compounds with concentrations greater than 1 percent of the total sum are displayed) for (A) fish muscle and (B) fish liver collected from different locations in 2023. PFSAs are denoted with warm pink/red hues, PFCAs with yellow to blue hues, and finally, precursors with grays and black. * Note the higher concentration of PFBA in Abiquiu fish liver samples.

The composition of PFAS in muscle samples was not indicative of PFAS composition in liver samples (Fig 5). In general, liver samples had a higher diversity of unique PFAS compounds (16 PFAS compounds) detected compared with muscle samples (9 PFAS compounds).

**Fig 6:**
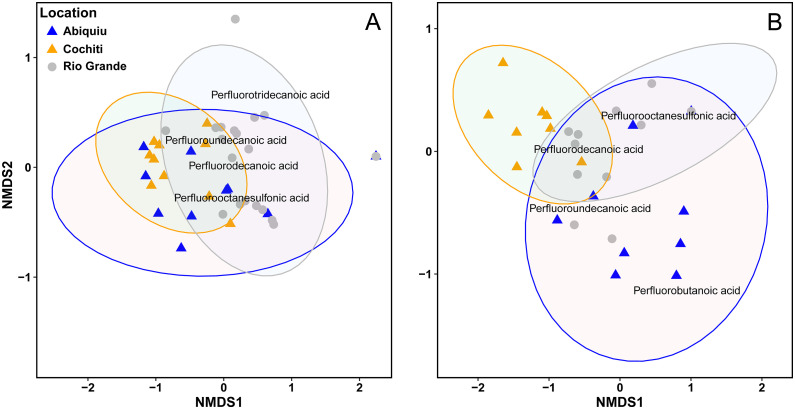
Nonmetric multidimensional scaling of PFAS results from fish (A) muscle and (B) liver from Abiquiu Reservoir, Cochiti Reservoir, and from the Rio Grande. Points and triangles represent the PFAS composition results (detects and non-detects) from each fish sample; axes are arbitrary. The four most detected PFAS for each sample type (muscle, liver) are displayed on each figure (A, B) relative to the location where they were detected. Muscle samples collected from the Rio Grande were significantly different than those collected from Cochiti Reservoir (p = 0.003; Fig 6A). For the liver samples, those collected from Abiquiu Reservoir were different from Cochiti Reservoir (p = 0.006), and both reservoirs were different from the Rio Grande samples (Abiquiu p = 0.003; Cochiti p = 0.027).

A NMDS analysis demonstrated a statistical difference in PFAS composition among sampling locations (Fig 6). For the muscle samples, only those collected from the Rio Grande were different than those collected from Cochiti Reservoir (p = 0.003; Fig 6A). For the liver samples, those collected from Abiquiu Reservoir were different from Cochiti Reservoir (p = 0.006), and both reservoirs were different from the Rio Grande samples (Abiquiu p = 0.003; Cochiti p = 0.027), with Abiquiu having some of the highest and most unique detection concentrations of PFBA (Fig 6B).

### δ^15^N and δ^13^C stable isotope values and correlation to PFAS

**Fig 7:**
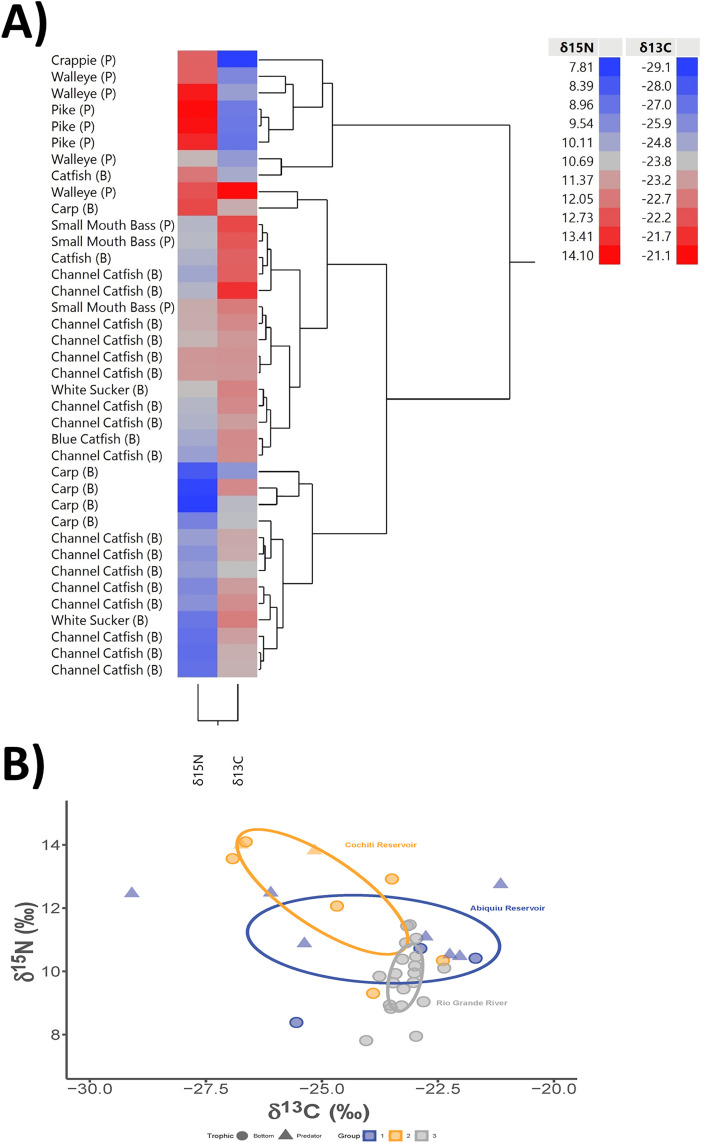
(A) Hierarchical clustering analysis of fish taxa versus δ^15^N and δ^13^C concentrations in liver samples. Fish-feeding strategy is represented with a (P) for predator or (B) for bottom-feeding next to the common name. Significance within the analysis was assessed by examining the dendrogram and the distances between clusters. The longer line segments suggest greater dissimilarity between clusters, indicating more significant separation between samples. (B) Visualization of Bayesian-based stable isotope standard ellipse areas (see Jackson et al. 2011) for fish within each location (Cochiti Reservoir, Abiquiu Reservoir, and the Rio Grande) in our sample [36]. Following Jackson et al. 2011 and a recent application by Dombrosky et al. 2020, we use small sample size corrected standard ellipse areas for our visualization of isotopic niche width [31,36]. Sample points are distinguished by shapes for trophic position and color for location.

Stable isotopes measured from fish liver samples revealed that enriched (i.e., contains increased amounts) δ^15^N values were predominantly observed in predatory fish, whereas enriched δ^13^C values were predominantly observed in bottom-feeding fish (Fig 7; Panel A; S1 Table). The variability in δ^13^C and δ^15^N fish liver values also matches other taxon-specific stable isotope data from 20th and 21st century fishes collected from various locations within the Rio Grande [31–33], including when correcting for tissue-specific discrimination [34,35]. Overall, nitrogen isotopes range between 7.8‰ in a bottom-feeding carp from the Rio Grande to 14.1‰ in a prey-feeding pike from Cochiti Reservoir. Carbon isotopes range between −29.1‰ in a white crappie from Abiquiu Reservoir to −21.1‰ in a walleye, also from Abiquiu Reservoir, but the majority of fishes in this sample average −23.8‰, which is indicative of more depleted 21st-century bottom-feeding carbon isotope values within the Rio Grande system.

Overall, predatory fish have enriched δ^15^N values while bottom feeding fish have enriched δ^13^C values. However, each sample location has locally dependent inputs influencing the underlying quantity, availability, and isotopic signal of food sources. Fishes from the Abiquiu Reservoir have similar and overlapping stable isotope niche areas as fishes from Cochiti Reservoir and the Rio Grande, but Cochiti Reservoir and the Rio Grande samples compared together show very little similarity in isotope niche area, even though they are co-located within the same river system.

**Fig 8:**
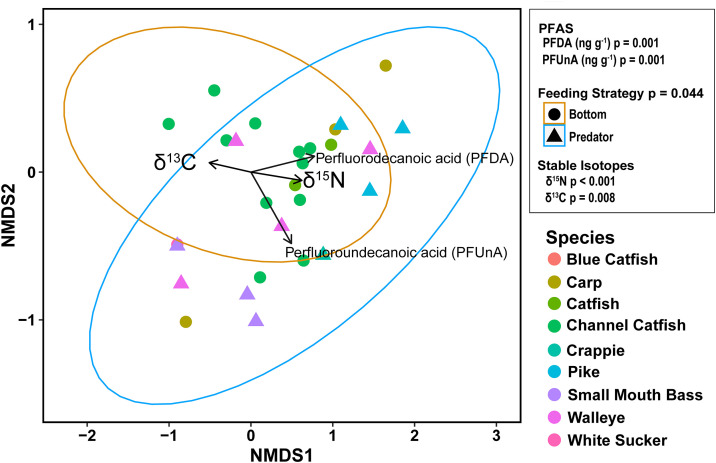
Nonmetric multidimensional scaling (NMDS) of PFAS composition in fish liver samples taken from Abiquiu Reservoir, Cochiti Reservoir, and the Rio Grande. Circles (bottom feeder) and triangles (predators) indicate different feeding strategies; colors denote taxon. Vector arrows show significant variables (PFAS and stable isotope data) and the direction of significant influence driving the distribution patterns in the NMDS (p < 0.05).

NMDS results were generated from two convergent solutions and two dimensions, with stress = 0.048. We assessed differences in PFAS composition in liver tissue between feeding strategies based on assigned categories (bottom and predator, p = 0.044) and data from stable isotope samples (δ^15^N, p < 0.001; δ^13^C, p = 0.008). Stable isotope values were displayed as vectors, where the length of the vector corresponds to the strength of the influence; longer vectors have stronger influences and shorter vectors are less significant (Fig 8). To investigate which PFAS driving distribution patterns, we used results from the simper analysis and displayed vectors of PFAS that showed the strongest influences on the distribution of points (p < 0.05) and were detected at all locations at least once; these PFAS included PFDA and PFUnA (Fig 8).

**Fig 9:**
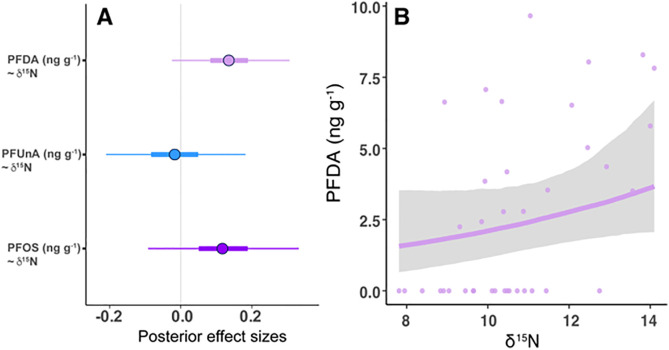
Results from Bayesian multivariate mixed model for fish liver samples collected at Abiquiu and Cochiti reservoirs and the Rio Grande. **(A)** Posterior means and 95% credible interval (CI) for the effect sizes (β estimate) of δ^15^N on perfluorodecanoic acid (PFDA), perfluoroundecanoic acid (PFUnA), and perfluorooctanesulfonic acid (PFOS). An effect size is considered strongly supported by the data if the CI for the effect size does not include zero (B) perfluorodecanoic acid (PFDA) concentrations and δ^15^N results; line shows median posterior estimates, and gray bands indicate 95 percent credible intervals.

We used a Bayesian multivariate mixed model analysis to further investigate relationships between δ^15^N values and PFAS of interest that were detected across all sites (PFDA, PFUnA, and PFOS), while also controlling for taxa. The estimated posterior mean effect of δ^15^N values on PFDA concentrations in fish liver samples was 0.14 with the 95% credible interval [−0.02, 0.31], narrowly crossing zero. The 90% credible interval excluded zero, indicating moderate but not strong evidence for a positive relationship. The positive relationship with PFDA concentrations and δ^15^N values shows that fish species that contained enriched δ^15^N values tended to have higher PFDA concentrations (Fig 9B). The estimated effect of δ^15^N values on PFOS concentrations was weaker but still positive, with a posterior mean of 0.12 (95% credible interval: −0.09 to 0.33). We found no evidence for an effect of δ^15^N values on PFUnA with a posterior mean of −0.02 (95% credible interval: −0.21 to 0.18) (Fig 9A).

## Discussion

Although originally thought to be nontoxic and noncarcinogenic, PFAS have drawn significant attention from government agencies and the public during the last decade due to their toxicological, carcinogenic, and environmental stability properties, even garnering the buzzworthy nickname of “forever chemicals.” In this study, we examined PFAS concentrations in different tissues of fish collected in northern New Mexico waterways. Our investigation into PFAS concentrations and composition in northern New Mexico is the first study to demonstrate a correlation between PFAS concentrations and compositions and stable isotope values within these bodies of water. The findings of this investigation provide significant information on the composition and concentrations of different PFAS chemicals associated with fish taxa, their feeding ecology, and a potential route of human exposure through the consumption of fish.

When we examined different fish tissue (muscle and liver) for the presence of 39 different PFAS compounds, we detected 9 different PFAS chemicals in fish muscle and 16 different PFAS chemicals in fish liver samples. Overall PFOS concentrations in liver samples were significantly higher than muscle samples, suggesting sequestration and accumulation by the liver. The predominant PFAS chemical that we observed in both muscle and liver samples was PFOS (C_8_HF_17_O_3_S), a long-chain PFAS compound. Liver samples overall had higher concentrations of PFOS when compared with muscle samples. When we examined the ratio between muscle and liver concentrations for PFOS, we noted that PFOS concentrations ranged from 1.13 to 350.1 (64.4 average) times higher in the liver samples. These findings are corroborated by a review from Khan et al. 2023 who noted higher PFAS concentrations in fish liver than muscle samples [37]. Within the review Khan et al. 2023 noted liver concentrations of PFOS ranged from 39.83 ng g^-1^ in spotted seatrout (*Cynoscion nebulosus*) up to 135 ng g^-1^ in dusky flathead (*Platycephalus fuscus*) [37].

We also noted that liver samples collected from Abiquiu had high concentrations of PFBA (C_4_HF_7_O_2_), which we did not find to the same extent in other locations examined. Toxicity of PFBA depends on the concentration but ranges from the generation of reactive oxygen species to developmental abnormalities [38]. The high concentration of PFBA within a specific location could be the result of multiple inputs, breakdown of parent PFAS compounds, or a specific-point source contamination within the environment [39,40]. Although specific point-source contaminations are well established, Abiquiu Reservoir is known for high boating traffic, is close to a heavily trafficked state highway (Hwy 84), and historically had an airstrip within proximity to the reservoir (Ghost Ranch Strip Airport). Langberg et al. 2022 examined the distribution of PFAS based on the source of contamination and concluded that higher concentrations of perfluoroalkanesulfonic acids (PFOS, PFDS, PFHxS) could be associated with lakes contaminated with AFFF, whereas higher concentrations of percent perfluoroalkyl carboxylic acids (PFNA, PFUnDA, PFDA, PFDoDA, PFHxA, PFTeDA, PFTrDA) were consistent with long-range atmospheric deposition in fish [41]. Further, Dasu et al. 2022 and Backe et al. 2013 also correlated AFFF with high concentrations of PFOS, PFOA, along with other long chain PFAS compounds, but noted that AFFF PFAS concentration and composition can vary and most AFFF compositions are proprietary which can make it hard to classify all PFAS compounds which are associated with AFFF [42,43]. Langberg et al. 2022 also noted that point source contamination was higher (5.0–272 ng g^-1^) compared with atmosphere transport (1.2–4.1 ng g^-1^) [41]. Within our investigation, we see a mixture of both perfluoroalkanesulfonic acids and perfluoroalkyl carboxylic acids, based on the findings from Langberg et al. 2022 we can suggest both point source contamination and atmospheric deposition; however, our total PFAS concentration in muscle samples is more in line with the results of atmosphere transport [41]. Additional studies are needed to confirm these hypotheses.

Travis et al. 2024 conducted water sampling in New Mexico in 2020 and 2021 to examine PFAS concentrations, including examining PFAS compounds in the Rio Chama and Rio Grande [7]. Within their investigation they noted PFAS concentrations that ranged from 1 to 155.5 ng L^-1^, including high detections of PFBS (93 ng L^-1^) in the Rio Grande downstream of Albuquerque [7]. Overall, PFAS concentrations within the Rio Chama ranged from approximately 8 ng L^-1^ to non-detects, depending on the sampling event and time of year [7]. Travis et al. 2024 also noted specific discharge sources into Northern New Mexico water ways, including municipal and fish hatchery into the Rio Chama and federal, municipal, and private domestic discharges into the Rio Grande above the Buckman Diversion [7]. Travis et al. 2024 also suggested that multiple facilities have the potential to handle PFAS with in the near-site watershed examined including, waste management and airports [7]. Overall, there are relatively low inputs into the Rio Chama when compared to more urban areas further downstream in the Rio Grande [7].

All predatory feeding fishes (collected only from Abiquiu and Cochiti reservoirs), including white crappie, walleye, and pike, had enriched δ^15^N values. Smallmouth bass, while also being prey-based feeders, were variable and had measured δ^15^N values that matched some bottom-feeding fishes. White crappies, walleye, pike, and smallmouth bass have diets that shift throughout their juvenile and adult life stages, but they include insects, larvae, other small fishes, invertebrates, amphibians, and other animals when opportunistically available. Unsurprisingly, predatory fish are enriched in δ^15^N due to their higher position in the trophic scale, but the generally elevated δ^15^N values within fish from both Abiquiu and Cochiti reservoirs suggest an underlying influence of anthropogenic nitrogen (such as wastewater) into these systems [31,33]. The anthropogenic structure of the Rio Grande, including the construction of both reservoirs, has shifted nutrient transfer from carbon 4 (C4) to carbon (C3) photosynthetic plants (carbon fixation pathways used by plants) across taxa, leading to a shift toward enriched C3-focused δ^13^C derived plant values in fish dietary signals [31,33].

The results of our investigation provide insight into the presence and distribution of PFAS compounds found in northern New Mexico, including the correlation that longer-chain PFAS compounds in liver tissue—such as PFDA (C_9_F_19_COOH) and PFUnA (C_10_F_21_COOH)—are associated with higher trophic levels (predatory fish) and nitrogen isotopic values. Within the liver tissue, both our NMDS analysis and Bayesian models showed moderate evidence for a positive nitrogen isotopic correlation with PFDA, whereas our NMDS analysis showed a significant correlation with PFUnA and PFDA. The difference likely stems from how these approaches handle the data. NMDS provides a non-parametric, distance-based assessment of overall compositional patterns. In contrast, our Bayesian model directly estimates the effect of δ^15^N on each compound individually while explicitly modeling the zero-inflated, left-skewed data and accounting for the shared variation among species through random effects. This more direct parametric modeling approach can reduce apparent associations if, for example, the relationship for PFUnA is confounded by species-level differences or by the distributional properties of the data. Thus, the discrepancy likely reflects the Bayesian model’s ability to disentangle these effects and isolate the direct association between δ^15^N and each individual PFAS compound. Still, the results suggest that these higher trophic-level fish are accumulating significant amounts of these long-chain PFAS chemicals, which could potentially have toxicological consequences for both fish and human health (through consumption) when exposed to high enough concentrations. In controlled feeding experiments, fish that consumed low-quality diets with higher plant-based protein amounts (instead of animal-based protein) had slower nitrogen isotopic turnover rates in their liver tissue [34]; however, nitrogen isotopic turnover rates in liver were faster than in muscle due to metabolic turnover. This result suggests that the quality of dietary protein that fishes consume could influence metabolic processes and their underlying isotopic values [44], but it is unclear if a similar process influences PFAS incorporation and concentration in liver tissue compared with muscle. The breakdown product of grease-proof coatings PFDA and PFUnA is primarily associated with food packaging and stain or grease protection for furniture and carpets [45,46]. PFDA has been demonstrated to have endocrine disruption properties [47] and has been associated with oxidative stress in zebra fish [48], whereas PFUnA has been demonstrated to cause hepatotoxicity at the lowest dose tested of 100 ng g^-1^ [49]. These reports suggest that PFDA and PFUnA are two PFAS compounds that can have toxicological ramifications on species exposed to these chemicals.

When mean muscle tissue concentrations in northern New Mexico waterways were compared with the United States, we noted that total PFAS (39 tested compounds) concentrations in fish in northern New Mexico within our investigation (2.02 ± 1.81 ng g^-1^) were lower than the mean tissue concentrations found in the United States 20.8 ng g^-1^ (13 tested compounds) [13]. We also noted that the sum of PFOS, PFOA, PFNA, and PFHxS in our investigation in northern New Mexico (1.30 ± 1.21 ng g^-1^) was lower than the 2 ng g^-1^ maximum concentration (sum of PFOS, PFOA, PFNA, and PFHxS) of fish on the market in the European Union, with approximately 70 percent of the fish examined in this study falling below these guidelines. Although the health consumption guidelines for fish vary from country to country (2 ng g^-1^ maximum concentration [sum of PFOS, PFOA, PFNA, and PFHxS] of fish on the market in the European Union compared with the Great Lakes Consortium for Fish Consumption of unrestricted consumption of fish should contain PFOS lower than 10 ng g^-1^), a metadata report by Langberg et al. (2024) suggests that fish exposed to pollution from diffuse sources could pose a risk to not only highly exposed individuals who are consuming vast amounts of fish but also all individuals who consume fish [19,50].

A field environment could contain multiple sources of PFAS input and contamination. We made significant efforts to limit PFAS contamination from equipment, including testing equipment for the presence of PFAS and not wearing known PFAS-laden personal care products or clothes during sampling events. Furthermore, we collected PFAS water blanks during sampling events to determine if contamination from existing atmospheric conditions could have skewed sample collections. Three PFAS water blanks collected along the Rio Grande contained one detectable PFAS compound, the PFAS compound was the same compound in all three-water blank and was PFPeA (ranged from 0.858–1.55 ng L^-1^), however, PFPeA was not detected in tissue samples within this investigation. Although we made every effort to limit any PFAS input during this investigation, PFAS have become ubiquitous in sampling equipment, including fishing gear. Additionally, the elevated water levels in northern New Mexico during 2023 sampling events could have increased inputs into the reservoirs and the Rio Grande. Within the waterways of northern New Mexico water levels tend to fluctuate depending on the time of the year. Both Abiquiu and Cochiti Reservoirs can have increased discharge during the spring, which was observed in 2023. Data from the United States Geological Survey suggested that the water discharge from Cochiti Reservoir was in line with previous release over the past 10 years, while the discharge from Abiquiu Reservoir (~5,000 ft^3^ -s) was higher than the previous past 10 years (~4,000 ft^3^ -s) [51]. It is hard to quantify if the increased water levels influenced PFAS concentrations within the reservoirs without water PFAS data. Although water levels were high in 2023, sampling events at Cochiti Reservoir occurred weeks after flooding, and nitrogen levels were within reported literature values [52]. Further, we did not determine the sex and age of the fish collected in this species, which could add to variations in PFAS concentration within tissue samples. Even though uncertainties exist within ecological studies, we believe that concentrations of PFAS detected within fish tissue collected in this study represent accurate PFAS levels within fish taxa in northern New Mexico waterways.

In the current study, we detected multiple PFAS compounds within tissue sampled from fish collected in northern New Mexico. As more information becomes available on the toxicological ramifications of PFAS exposures, new information is needed to examine the presence of these chemicals in ecological settings. Fish taxa are a major group of interest for PFAS exposure due to human health concerns (via consumption) and the ability of fish to be used as a sentinel taxon to evaluate potential environmental contamination in waterways. We determined that PFAS accumulated in higher concentrations in liver samples and that specific PFAS compounds are associated with upper trophic level fish and higher δ^15^N stable isotopic values. Future work is needed to determine if the concentrations of PFAS compounds found in this study pose a concern for fish and human health. Additional information on point-source inputs, age, and sex of fish taxa could add significant information on PFAS accumulation within these waterways; however, within this study, we demonstrated the detection of PFAS compounds in almost all fish tissues collected in northern New Mexico waterways, indicating their ubiquity in the aquatic environment.

## Supporting information

S1 Table Fish specifications including C:N ratios. (CSV)
